# Construction and validation of a predictive model for postoperative pneumonia in elderly patients with hip fractures: A multicenter retrospective cohort study

**DOI:** 10.1097/MD.0000000000050255

**Published:** 2026-08-14

**Authors:** Daxue Zhang, Jian Kang, Yongli Zhang, Shiwei Yang, Xuchun Li

**Affiliations:** a Department of Orthopaedics, The First Affiliated Hospital of Shenzhen University/ Shenzhen Second People’s Hospital, Shenzhen, Guangdong, China; b Teaching Office, The First Affiliated Hospital of Shenzhen University/ Shenzhen Second People’s Hospital, Shenzhen, Guangdong, China.

**Keywords:** aged, hip fractures, nomogram, postoperative pneumonia, risk prediction model

## Abstract

To identify independent risk factors for postoperative pneumonia (POP) in elderly patients with hip fractures and to develop a nomogram for predicting its occurrence, we conducted a multicenter retrospective cohort study of elderly patients (≥ 60 years) who underwent hip fracture surgery at 3 hospitals in Shenzhen, China. Data were collected from medical records. Univariate and multivariate logistic regression analyses, combined with least absolute shrinkage and selection operator regression, were used to identify independent risk factors for POP. A nomogram prediction model was constructed. Model performance was evaluated using the area under the receiver operating characteristic curve for discrimination and the Hosmer–Lemeshow test for calibration. Internal validation was performed using the bootstrap method with 500 resamples. A total of 976 patients were included, with a mean age of 77.46 ± 8.85 years; 73.26% were female. POP occurred in 64 patients (6.56%). Multivariable logistic regression analysis identified a higher white blood cell count, a lower lymphocyte count, a lower albumin level, a history of chronic obstructive pulmonary disease or heart failure, and postoperative intensive care unit admission as independent risk factors for POP (all *P* < .05). Age was also an independent predictor. The model demonstrated an area under the curve of 0.795 for the development cohort and 0.792 for the internal validation cohort. The Hosmer–Lemeshow test showed good calibration (*P* = .9695 and 0.3644, respectively). A nomogram based on 7 readily available clinical factors can predict POP in elderly hip fracture patients with moderate accuracy. This internally validated model may assist clinicians in identifying high-risk patients, but further external validation is required before clinical implementation.

## 1. Introduction

Due to accelerated population aging, the incidence of hip fractures in the population has been increasing annually. The incidence rate of hip fractures in individuals aged ≥ 60 years exceeds 214.89 per 100,000.^[1]^ It is predicted that by the middle of this century, the global number of hip fractures will surpass 5 million, with approximately half of them occurring in Asia.^[2]^ The primary approach in managing hip fracture patients is surgical treatment, which aims to enable early mobilization and reduce bed rest complications.^[3]^ However, more than one-third of hip fracture patients cannot regain independent mobility within 1 year after surgery,^[4]^ with a high mortality rate of 17 to 27% within 1 year and exceeding 40% within 5 years.^[5]^

Research^[6,7]^ has indicated that one of the main causes of postoperative mortality in elderly hip fracture patients is pneumonia. The increased susceptibility to postoperative pneumonia (POP) in this population can be attributed to several factors, including declined physical function, multiple comorbidities, an age-related decline in immune competence, prolonged bed rest, and reduced energy metabolism following surgery.^[8,9]^ Reported incidence rates of POP range from 4.1 to 15%.^[10–12]^

Given the high morbidity and mortality associated with POP, effective perioperative management is crucial. For instance, interdisciplinary care pathways incorporating comprehensive geriatric assessment have been shown to reduce in-hospital mortality and improve functional recovery in elderly hip fracture patients.^[13]^ However, despite these interventions, the risk of POP remains substantial. Furthermore, even with optimized orthogeriatric management, patients continue to face significant risks, including loss of functional autonomy and increased 1-year mortality, which are closely linked to postoperative complications.^[14]^ Timely and accurate identification of high-risk individuals could enable clinicians to implement more intensive, personalized preventive measures, thereby potentially reducing the incidence of POP and improving patient outcomes. The development of a robust clinical prediction tool that synthesizes multiple risk factors into an individualized risk estimate represents a distinct methodological paradigm. Such a tool could facilitate risk stratification and guide clinical decision-making. However, multicenter-based predictive models specifically designed for POP in this vulnerable population remain limited.

Therefore, the primary objective of this multicenter retrospective cohort study was to develop and internally validate a nomogram-based predictive model for POP in elderly patients undergoing hip fracture surgery. By integrating readily available preoperative and perioperative clinical parameters, we aimed to create a practical tool that can assist clinicians in the early identification of high-risk patients, ultimately contributing to more targeted and effective perioperative care.

## 2. Methods

### 2.1. Population

This was a multicenter retrospective cohort study. We consecutively enrolled elderly patients with hip fractures admitted to Shenzhen Second People’s Hospital between January 1, 2021, and June 30, 2023, Shenzhen Pingle Orthopedics Hospital from January 1, 2021, to December 31, 2022, and Peking University Shenzhen Hospital from January 1, 2021, to June 30, 2022. The study was approved by the Ethics Committee of Shenzhen Second People’s Hospital (Ethics approval number: 20210620213357012-GZ2022) and registered with the Chinese Clinical Trial Registration Center (registration number: ChiCTR2100047560). Given the retrospective nature of this study, data anonymization was conducted in accordance with relevant laws, regulations, and policies. The Ethics Committee of Shenzhen Second People’s Hospital reviewed the study protocol and waived the need for patients’ informed consent due to the nature of the study.

#### 2.1.1. Inclusion criteria

X-ray diagnosis of femoral neck, intertrochanteric, or subtrochanteric fracture upon admission; age ≥ 60 years.

#### 2.1.2. Exclusion criteria

Preoperative pneumonia; fracture known for more than 3 weeks before hospital admission; pathological fracture due to malignant tumor metastasis; lack of surgical treatment; presence of blood system diseases; severe heart, lung, or renal insufficiency (defined as New York Heart Association class III–IV, requiring home oxygen therapy or having a history of chronic kidney disease [CKD] stage 4–5); multiple-site surgery; secondary fracture; incomplete data (defined as missing > 10% of key variables); in-hospital mortality (excluded to avoid survival bias, as these patients did not have the full postoperative period to develop POP).

### 2.2. Handling of missing data

For continuous variables following a normal distribution, missing values were imputed using the mean. For skewed continuous variables, missing values were imputed using the median. For categorical variables, missing values were imputed using the mode.

### 2.3. Definition of pneumonia

The diagnostic criteria for POP were based on the Chinese Guidelines for the Diagnosis and Treatment of Hospital-acquired Pneumonia and Ventilator-associated Pneumonia (2018 edition).^[15]^ These include chest X-ray or computed tomography revealing new or progressive infiltrating shadows, solid shadows, or ground-glass shadows, along with 2 or more of the following 3 clinical symptoms establishing a clinical diagnosis: fever, body temperature > 38°C; purulent airway secretions; peripheral blood white blood cell (WBC) count > 10 × 10^9^/ L or < 4 × 10^9^/ L. Diagnosis was based on routine clinical documentation by the attending physicians; however, given that all 3 participating hospitals are tertiary referral centers adhering to the same national guidelines, the diagnostic criteria were applied consistently across sites. POP, the outcome indicator in this study, refers to infections occurring 24 hours after surgery during the patient’s hospitalization.

### 2.4. Exposed variable

Baseline data: patient characteristics, including age, sex, body mass index, smoking history, fracture type, preoperative blood transfusion, and preoperative comorbidities (hypertension, diabetes mellitus, stroke, coronary heart disease, chronic bronchitis, chronic obstructive pulmonary disease [COPD], CKD, heart failure [HF], and cognitive impairment). Preoperative laboratory examination: WBC count, neutrophil count, lymphocyte count, hemoglobin, albumin, albumin-globulin ratio (AGR), urea nitrogen, and serum creatinine. Intraoperative operation and anesthesia data: American Society of Anesthesiologists (ASA) classification, operation duration, operation method, anesthesia method, intraoperative blood loss, fluid intake, etc. Postoperative admission to the intensive care unit (ICU) and mechanical ventilation.

### 2.5. Statistical analysis

Continuous variables following a normal distribution (or nearly normal distribution) are presented as mean ± standard deviation (*χ̄* ± s). Intergroup comparisons were conducted using analysis of variance and independent sample *t*-tests. Skewed continuous data are represented by median (interquartile range) (M [Q1–Q3]), and between-group comparisons were performed using Kruskal–Wallis tests and Wilcoxon tests. Categorical variables are expressed as frequency (percentage) (n [%]), and between-group comparisons were assessed using the *χ*^2^ test or Fisher exact test. To avoid the limitations of univariate preselection and to address multicollinearity, we employed a structured modeling approach. First, all candidate variables were entered into a least absolute shrinkage and selection operator (LASSO) regression for initial variable selection. LASSO regression is particularly suited for prediction modeling as it shrinks regression coefficients and performs automatic variable selection. The tuning parameter *λ* was selected using 10-fold cross-validation based on the minimum criteria (lambda.min). Variables with nonzero coefficients in the LASSO model were then included in a multivariable logistic regression analysis using backward stepwise selection based on the Akaike information criterion. Model performance evaluation included assessment of the area under the receiver operating characteristic curve (area under the curve [AUC]) and model differentiation. The optimal probability threshold for calculating sensitivity and specificity was determined using the Youden index. Calibration of the model was assessed using calibration plots and the Hosmer–Lemeshow test. The clinical benefit of the model was evaluated through decision curve analysis (DCA). A nonparametric resampling method (bootstrap) with 500 resampling iterations was used for the internal validation. R (The R Foundation; http://www.r-project.org; version 4.2.0) and EmpowerStats 4.1 (www.empowerstats.net; X&Y Solutions, Inc.) were used for statistical analysis. Bilateral tests were used in all hypothetical tests, and P < .05 was considered statistically significant.

## 3. Results

### 3.1. Baseline characteristics of the patients

**Figure 1.**
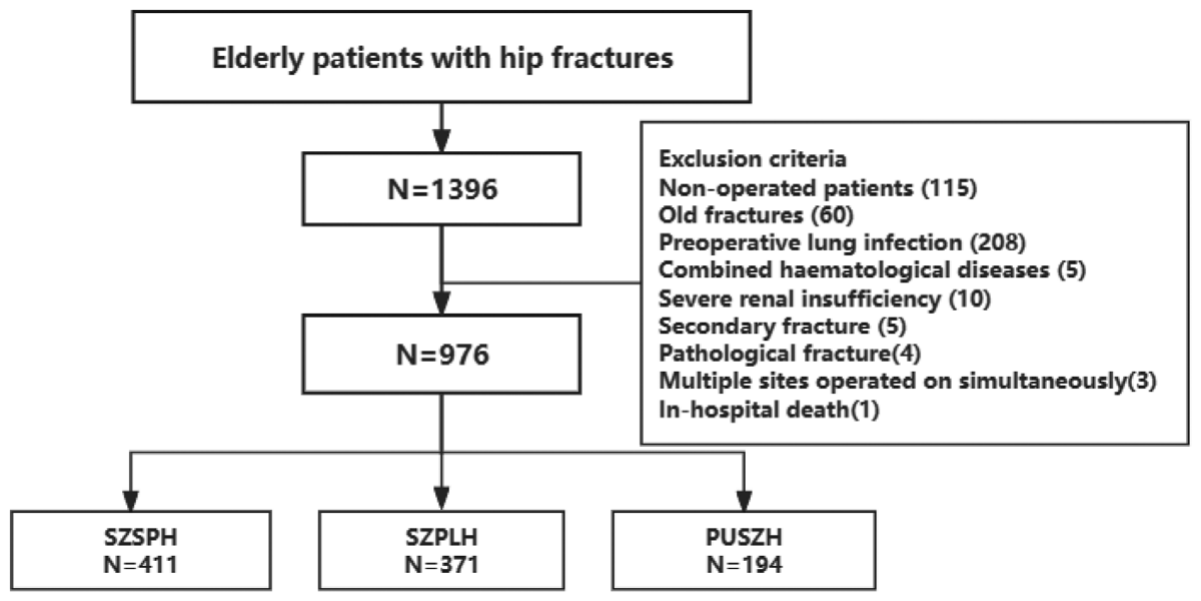
Flow chart of patient selection. N = number of patients, PUSZH = Peking University Shenzhen Hospital, SZPLH = Shenzhen Pingle Orthopedics Hospital, SZSPH = Shenzhen Second People’s Hospital.

Our study initially included 1396 subjects. Among them, 115 nonsurgical patients, 60 cases of old fractures, 208 cases of preoperative pneumonia, 5 cases of combined blood system diseases, 10 cases of severe renal insufficiency, 5 cases of secondary fractures, 4 cases of pathological fractures, 9 cases with incomplete data, 3 cases of multisite surgeries, and 1 case of in-hospital death were excluded. After exclusions, 976 elderly patients were included as study subjects. The study subject selection process is illustrated in Figure 1.

The clinical data of 411 elderly patients with hip fractures from Shenzhen Second People’s Hospital, 371 patients from Shenzhen Pingle Orthopedics Hospital, and 194 patients from Peking University Shenzhen Hospital were included in this study. The average age of the included patients was 77.46 ± 8.85 years, 73.26% were females, and the incidence of POP was 6.56%. The results of the intergroup comparison (Table 1) showed that there were statistically significant differences in smoking history, preoperative comorbidities (stroke, coronary heart disease, chronic bronchitis, and CKD), number of comorbidities, WBC count, neutrophil count, lymphocyte count, hemoglobin, albumin, leukocyte ratio, serum creatinine, ASA classification, operation duration, operation method, intraoperative blood loss, postoperative ICU admission, and mechanical ventilation (All *P* < .05).

**Table 1:** Comparison of characteristic data between groups of different hospitals.

Variables	Total (n = 976)	Hospitals			Statistic	*P* value
		SZSPH (n = 411)	SZPLH (n = 371)	PUSZH (n = 194)		
Age (yrs)	77.46 ± 8.85	77.92 ± 8.99	77.16 ± 8.89	77.08 ± 8.47	*F* = 0.94	.391
Sex, n (%)					*χ*^2^ = 0.62	.735
Male	261 (26.74)	114 (27.74)	94 (25.34)	53 (27.32)		
Female	715 (73.26)	297 (72.26)	277 (74.66)	141 (72.68)		
BMI (kg/m^2^)	22.19 ± 3.39	22.09 ± 3.31	20.75 ± 4.36	22.40 ± 3.56	*F* = 0.70	.496
Smoking, n (%)	63 (6.45)	31 (7.54)	14 (3.77)	18 (9.28)	*χ*^2^ = 7.78	.020
Fracture type, n (%)					*χ^2^* = 0.29	.865
Fracture of femoral neck	589 (60.35)	250 (60.83)	220 (59.30)	119 (61.34)		
Intertrochanteric fracture	387 (39.65)	161 (39.17)	151 (40.70)	75 (38.66)		
Transfusion, n (%)	93 (9.53)	36 (8.76)	36 (9.70)	21 (10.82)	*χ*^2^ = 0.67	.714
Complications, n (%)					*χ*^2^ = 27.14	< .001
0	182 (18.65)	72 (17.52)	84 (22.64)	26 (13.40)		
1–2	534 (54.71)	206 (50.12)	221 (59.57)	107 (55.15)		
≥3	260 (26.64)	133 (32.36)	66 (17.79)	61 (31.44)		
Hypertension, n (%)	532 (54.51)	236 (57.42)	199 (53.64)	97 (50.00)	*χ*^2^ = 3.11	.211
DM, n (%)	268 (27.46)	118 (28.71)	93 (25.07)	57 (29.38)	*χ*^2^ = 1.75	.417
Stroke, n (%)	276 (28.28)	119 (28.95)	84 (22.64)	73 (37.63)	*χ*^2^ = 14.27	< .001
CHD, n (%)	141 (14.45)	77 (18.73)	36 (9.70)	28 (14.43)	*χ*^2^ = 12.87	.002
Chronic bronchitis, n (%)	60 (6.15)	18 (4.38)	34 (9.16)	8 (4.12)	*χ*^2^ = 9.456	.009
COPD, n (%)	105 (10.76)	49 (11.92)	33 (8.89)	23 (11.86)	*χ*^2^ = 2.17	.339
CKD, n (%)	52 (5.33)	30 (7.30)	12 (3.23)	10 (5.15)	*χ*^2^ = 6.40	.041
HF, n (%)	36 (3.69)	21 (5.11)	10 (2.70)	5 (2.58)	*χ*^2^ = 4.04	.133
Cognitive impairment, n (%)	43 (4.41)	19 (4.62)	12 (3.23)	12 (6.19)	*χ*^2^ = 2.71	.257
WBC count (*10^9^/L)	8.79 ± 2.72	9.00 ± 2.85	8.88 ± 2.72	8.17 ± 2.33	*F* = 6.45	.002
Neutrophil count (*10^9^/L)	6.49 (5.00–8.39)	6.67 (5.06–8.58)	6.70 (5.06–8.75)	6.08 (4.59–7.19)	*H* = 6.18	.013
Lymphocyte count (*10^9^/L)	1.14 (0.90–1.50)	1.15 (0.92–1.46)	1.10 (0.80–1.50)	1.21 (0.96–1.60)	*H* = 12.40	< .001
Hemoglobin (g/L)	116.06 ± 17.50	117.66 ± 17.57	115.19 ± 16.79	114.34 ± 18.48	*F* = 3.13	.044
Albumin (g/L)	38.21 ± 4.05	38.57 ± 4.21	38.87 ± 3.87	36.18 ± 3.36	*F* = 33.02	< .001
AGR	1.34 ± 0.24	1.37 ± 0.25	1.34 ± 0.24	1.29 ± 0.21	*F* = 7.48	< .001
Urea nitrogen (mmol/L)	6.10 (4.90–7.70)	6.30 (5.00–7.81)	5.90 (4.70–7.50)	6.21 (4.93–7.77)	*H* = 4.36	.113
Serum creatinine (umol/L)	66.0 (55.0–83.0)	65.2 (53.2–79.25)	69.0 (58.0–85.0)	64.0 (54.0–78.0)	*H* = 11.33	.003
ASA classification, n (%)					*χ*^2^ = 81.75	< .001
≤Ⅱ	521 (53.38)	150 (36.50)	240 (64.69)	131 (67.53)		
≥Ⅲ	455 (46.62)	261 (63.50)	131 (35.31)	63 (32.47)		
Operation duration (min)	89.40 ± 29.72	94.39 ± 29.54	84.35 ± 32.58	88.51 ± 21.67	*F* = 11.49	< .001
Operation method, n (%)					*χ*^2^ = 15.58	.004
Total hip replacement	456 (46.72)	182 (44.28)	184 (49.60)	90 (46.39)		
Femoral head replacement	126 (12.91)	72 (17.52)	39 (10.51)	15 (7.73)		
Internal fixation	394 (40.37)	157 (38.20)	148 (39.89)	89 (45.88)		
Anesthesia method, n (%)					*χ*^2^ = 199.15	< .001
General anesthesia	388 (39.75)	254 (61.80)	47 (12.67)	87 (44.85)		
Intraspinal anesthesia	588 (60.25)	157 (38.20)	324 (87.33)	107 (55.15)		
Intraoperative blood loss (mL)	200 (100–300)	200 (100–300)	200 (100–300)	100 (50–200)	*H* = 88.44	< .001
Intraoperative fluid intake (mL)	1205.20 ± 431.62	1222.02 ± 446.52	1208.36 ± 479.91	1163.53 ± 271.43	*F* = 1.23	.294
ICU admission, n (%)	43 (4.41)	9 (2.19)	20 (5.39)	14 (7.22)	*χ*^2^ = 9.29	.010
Mechanical ventilation, n (%)	20 (2.05)	5 (1.22)	5 (1.35)	10 (5.15)	*χ*^2^ = 11.65	.003
POP, n (%)	64 (6.56)	25 (6.08)	24 (6.47)	15 (7.73)	*χ*^2^ = 0.59	.744

AGR = albumin-globulin ratio, ASA = American Society of Anesthesiologists, BMI = body mass index, CHD = coronary heart disease, CKD = chronic kidney disease, COPD = chronic obstructive pulmonary disease, DM = diabetes mellitus, HF = heart failure, ICU = intensive care unit, n = number of patients, POP = postoperative pneumonia, PUSZH = Peking University Shenzhen Hospital, SZPLH = Shenzhen Pingle Orthopedics Hospital, SZSPH = Shenzhen Second People’s Hospital, WBC = white blood cell.

### 3.2. Univariate analysis

Univariate analysis (Table 2) showed significant differences in age, sex, preoperative blood transfusion, number of comorbidities, stroke, chronic bronchitis, COPD, CKD, HF, WBC count, neutrophil count, lymphocyte count, hemoglobin, albumin, AGR, urea nitrogen, serum creatinine, ASA classification, surgical method, intraoperative fluid intake, ICU admission, and mechanical ventilation among the groups (all *P* < .05).

**Table 2:** Univariate analysis of POP.

Variables	No POP (n = 912)	POP (n = 64)	Statistic	*P* value
Age (yrs)	77.46 ± 8.85	83.27 ± 8.44	*t* = −5.51	< .001
Sex, n (%)			*χ*^2^ = 6.74	.009
Male	235 (25.77)	26 (40.62)		
Female	677 (74.23)	38 (59.38)		
BMI (kg/m^2^)	22.24 ± 3.36	21.44 ± 3.78	*t* = 1.41	.160
Smoking, n (%)	55 (6.03)	8 (12.50)	*χ*^2^ = 3.14	.076
Fracture type, n (%)			*χ*^2^ = 2.21	.137
Fracture of femoral neck	556 (60.96)	33 (51.56)		
Intertrochanteric fracture	356 (39.04)	31 (48.44)		
Transfusion, n (%)	81 (8.88)	12 (18.75)	*χ*^2^ = 6.76	.009
Complications, n (%)			*χ*^2^ = 19.72	< .001
0	181 (19.85)	1 (1.56)		
1–2	500 (54.82)	34 (53.12)		
≥3	231 (25.33)	29 (45.31)		
Hypertension, n (%)	495 (54.28)	37 (57.81)	*χ*^2^ = 0.30	.583
DM, n (%)	248 (27.19)	20 (31.25)	*χ*^2^ = 0.49	.482
Stroke, n (%)	250 (27.41)	26 (40.62)	*χ*^2^ = 5.15	.023
CHD, n (%)	127 (13.93)	14 (21.88)	*χ*^2^ = 3.06	.080
Chronic bronchitis, n (%)	49 (5.37)	11 (17.19)	*χ*^2^ = 12.49	< .001
COPD, n (%)	85 (9.32)	20 (31.25)	*χ*^2^ = 29.96	< .001
CKD, n (%)	43 (4.71)	9 (14.06)	*χ*^2^ = 8.59	.003
HF, n (%)	29 (3.18)	7 (10.94)	*χ*^2^ = 8.07	.005
Cognitive impairment, n (%)	40 (4.39)	3 (4.69)	*χ*^2^ = 0.00	1.000
WBC count (*10^9^/L)	8.73 ± 2.64	9.69 ± 3.64	*t* = −2.08	.042
Neutrophil count (*10^9^/L)	6.45 (4.96–8.31)	7.31 (5.83–9.59)	*Z* = −2.48	.013
Lymphocyte count (*10^9^/L)	1.15 (0.90–1.50)	0.97 (0.75–1.24)	*Z* = −3.52	< .001
Hemoglobin (g/L)	116.42 ± 17.31	110.92 ± 19.48	*t* = 2.43	.015
Albumin (g/L)	38.31 ± 4.03	36.76 ± 4.13	*t* = 2.98	.003
AGR	1.35 ± 0.24	1.27 ± 0.26	*t* = 2.63	.009
Urea nitrogen (mmol/L)	6.10 (4.80–7.60)	7.10 (5.48–9.11)	*Z* = −3.30	< .001
Serum creatinine (umol/L)	65.65 (55.00–82.00)	75.00 (62.77–102.25)	*Z* = −3.29	.001
ASA classification, n (%)			*χ*^2^ = 24.68	< .001
≤Ⅱ	506 (55.48)	15 (23.44)		
≥Ⅲ	406 (44.52)	49 (76.56)		
Operation duration (min)	89.68 ± 29.80	85.52 ± 28.41	*t* = 1.08	.280
Operation method, n (%)			*χ*^2^ = 11.46	.003
Total hip replacement	436 (47.81)	20 (31.25)		
Femoral head replacement	110 (12.06)	16 (25.00)		
Internal fixation	366 (40.13)	28 (43.75)		
Anesthesia method, n (%)			*χ*^2^ = 1.38	.240
General anesthesia	367 (40.24)	21 (32.81)		
Intraspinal anesthesia	545 (59.76)	43 (67.19)		
Intraoperative blood loss (mL)	200 (100–300)	200 (100–200)	*Z* = −0.93	.354
Intraoperative fluid intake (mL)	1213.73 ± 437.54	1083.66 ± 314.12	*t* = 2.34	.020
ICU admission, n (%)	30 (3.29)	13 (20.31)	*χ*^2^ = 37.21	< .001
Mechanical ventilation, n (%)	15 (1.64)	5 (7.81)	*χ*^2^ = 8.47	.004

AGR = albumin-globulin ratio, ASA = American Society of Anesthesiologists, BMI = body mass index, CHD = coronary heart disease, CKD = chronic kidney disease, COPD = chronic obstructive pulmonary disease, DM = diabetes mellitus, HF = heart failure, ICU = intensive care unit, n = number of patients, POP = postoperative pneumonia, WBC = white blood cell.

### 3.3. LASSO regression screening variables

**Figure 2.**
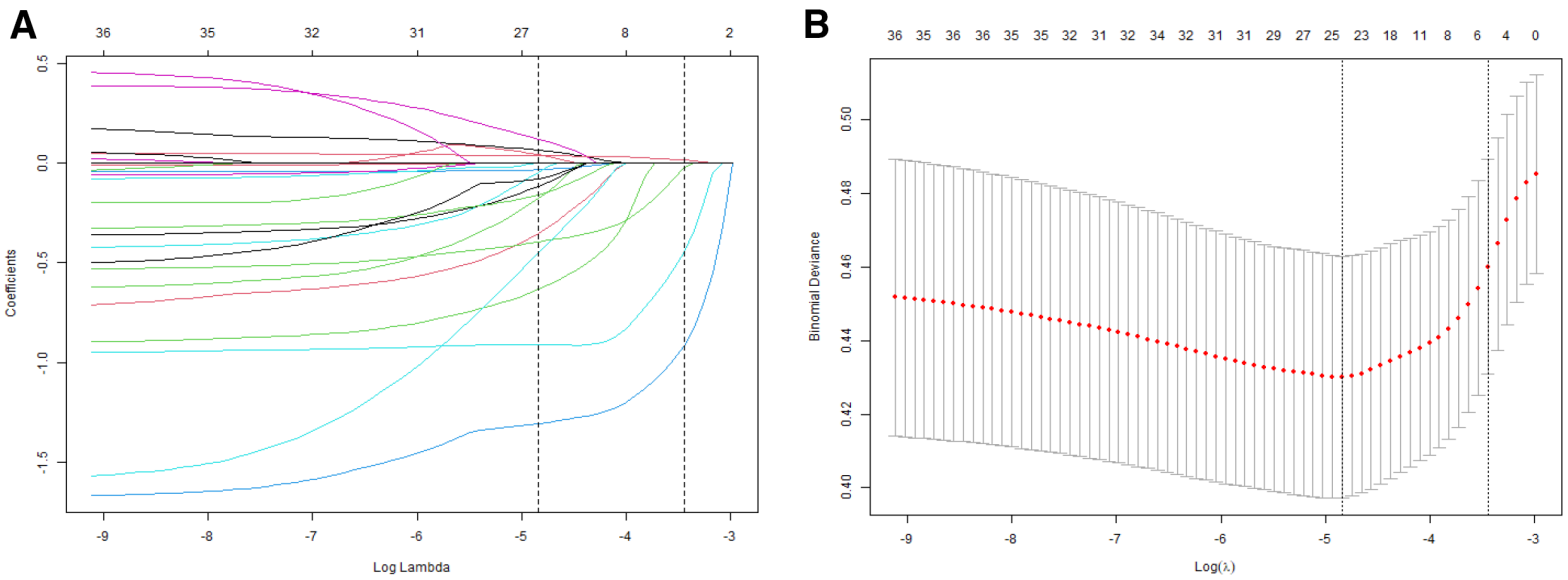
The potential risk factors were selected using LASSO regression. LASSO = least absolute shrinkage and selection operator.

To avoid multicollinearity among variables included in multivariate logistic regression analysis, LASSO regression was used to screen variables with *P* < .05 in the univariate analysis. Furthermore, although the intergroup comparison results showed no statistically significant differences in smoking, it was included in the LASSO regression. The results (Fig. 2) show that *λ* is the lambda. min (0.0079), a total of 17 variables were screened, including age, sex, smoking, stroke, chronic bronchitis, COPD, CKD, HF, number of complications, WBC count, lymphocyte count, albumin level, AGR, ASA classification, operation method, intraoperative fluid intake, and postoperative ICU admission.

### 3.4. Multivariate logistic regression analysis

According to the LASSO regression analysis, 17 variables, including age, sex, smoking, stroke, chronic bronchitis, COPD, CKD, HF, number of complications, WBC count, lymphocyte count, albumin, AGR, ASA classification, operation mode, intraoperative fluid intake, and postoperative ICU admission, were included in binary logistic regression analysis (likelihood ratio). The results (Table 3) showed that age, WBC count, lymphocyte count, albumin, COPD, HF, and postoperative ICU admission were independent risk factors for POP in elderly patients with hip fracture (all *P* < .05).

**Table 3:** Multivariable logistic regression of predictors for POP.

Regressor variable	*B*	SE	Wald *χ*^2^	OR	95% CI	*P* value
Constant	−4.951	2.095	5.586	0.007	-	.018
Age	0.057	0.018	9.872	1.059	1.022–1.098	.002
COPD	1.372	0.319	18.437	3.942	2.108–7.372	< .001
HF	1.118	0.482	5.382	3.057	1.189–7.859	.020
WBC count	0.127	0.047	7.195	1.135	1.035–1.245	.007
Lymphocyte count	−0.758	0.315	5.789	0.469	0.253–0.869	.016
Albumin	−0.083	0.034	5.980	0.920	0.861–0.984	.014
ICU admission	1.573	0.396	15.754	4.821	2.217–10.482	< .001

CI = confidence interval, COPD = chronic obstructive pulmonary disease, HF = heart failure, ICU = intensive care unit, OR = odds ratio, POP = postoperative pneumonia, SE = standard error, WBC = white blood cell.

### 3.5. Model and nomogram development

**Figure 3.**
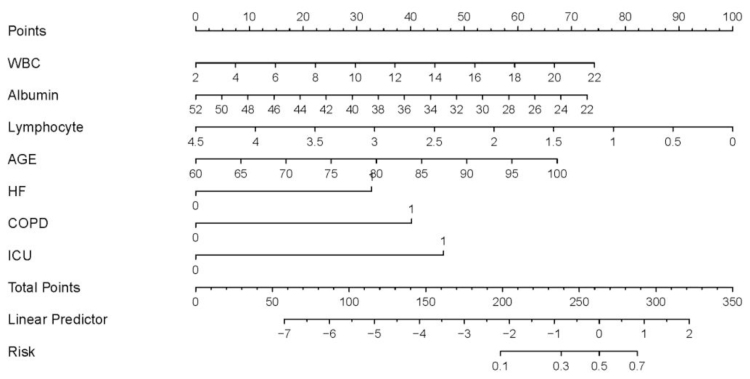
Nomogram for predicting the probability of postoperative pneumonia in elderly patients with hip fractures. COPD = chronic obstructive pulmonary disease, HF = heart failure, ICU = intensive care unit, WBC = white blood cell.

A multicenter predictive model was established for POP in elderly patients with hip fractures. Logistic regression analysis was conducted, incorporating several factors such as age, COPD, HF, leukocyte count, lymphocyte count, albumin level, and postoperative admission to the ICU. The formula of the model was derived as follows: The model formula was *Z* = −4.951 + 0.057*(age) + 1.372*(COPD) + 1.118*(HF) + 0.127*(WBC) − 0.758*(lymphocyte) − 0.083*(albumin) + 1.573*(ICU). A visual presentation of the simplified nomogram is shown in Figure 3.

### 3.6. Model validation

**Figure 4.**
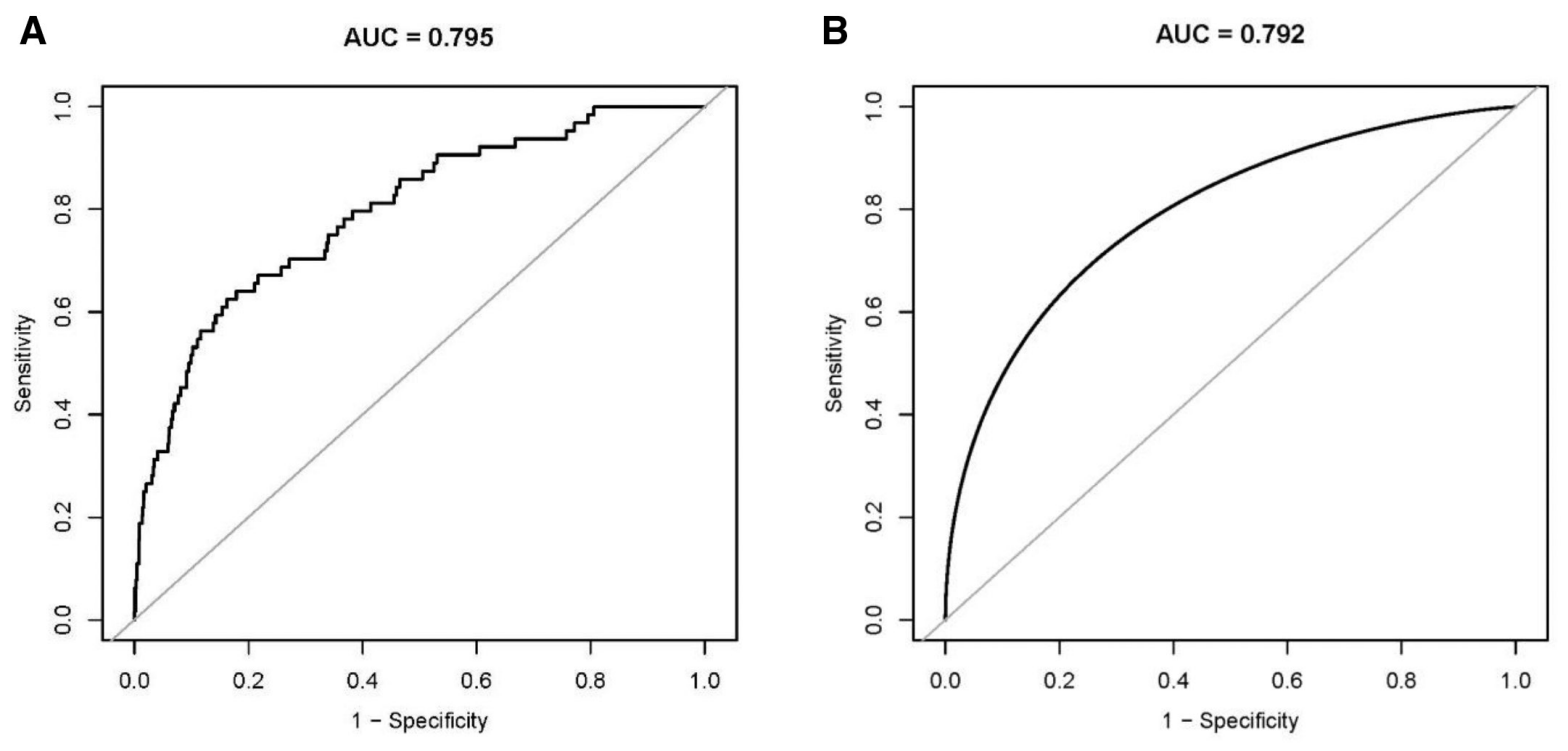
ROC curves for validating the discrimination power of the nomogram. AUC = area under the curve, ROC = receiver operating characteristic.

**Figure 5.**
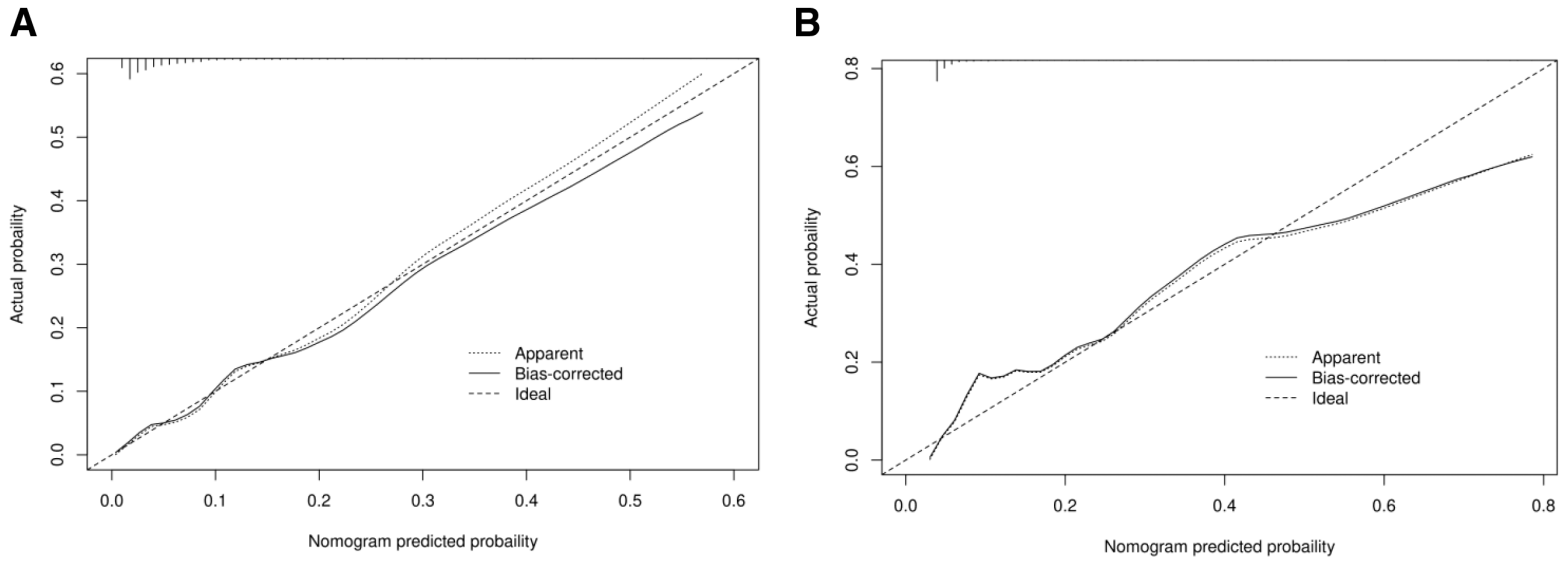
Calibration plot. The calibration curve showed good concordance between predicted probability and actual probability. (A) Modeling group; (B) internal verification group.

**Figure 6.**
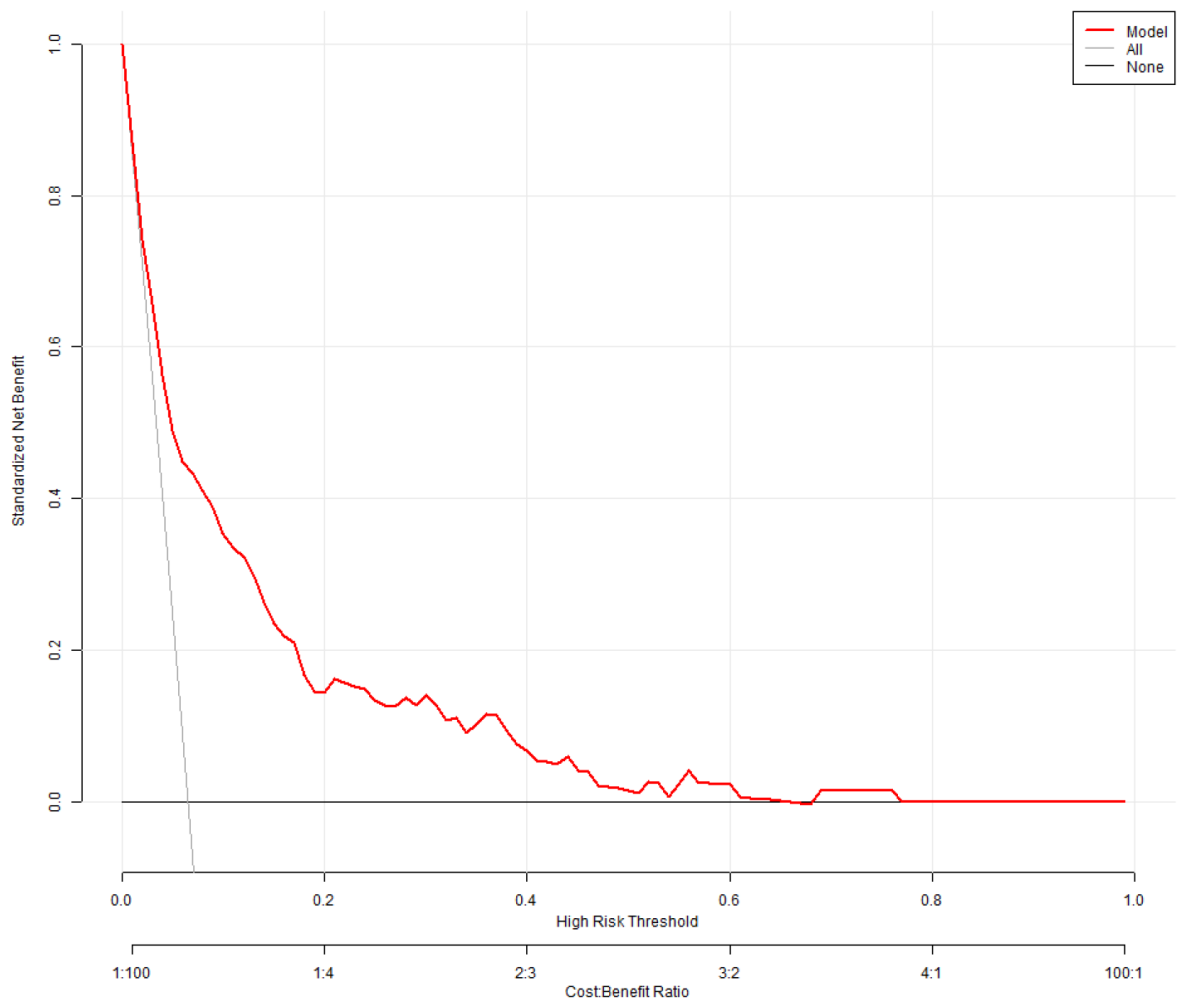
Decision curve plot. In the DCA, the nomogram was compared with the null model for its added value, which showed that the nomogram was applicable when thresholds were in the range between 1.75% and 65.38%, due to the net benefit. DCA = decision curve analysis.

The differentiation results (Fig. 4A) demonstrated an AUC value of 0.795 (95% confidence interval [CI]: 0.763–0.853) for this model. Using the optimal probability threshold determined by the Youden index, the model achieved a sensitivity of 62.5% and a specificity of 83.8%. The Hosmer–Lemeshow test for calibration showed a *χ*^2^ value of 2.3222 with a corresponding *P* value of .9695 (Fig. 5A). The internal verification results (Fig. 4B) showed that the AUC value was 0.792 (95% CI: 0.732–0.848), indicating that the model had a moderate discriminatory ability. The Hosmer–Lemeshow test for calibration showed a *χ*^2^ value of 8.7435 with a corresponding *P* value of .3644 (Fig. 5B), which indicated that there was no statistical significance between the predicted probability and the actual incidence. DCA curve analysis indicated that the maximum net benefit threshold probability of the model ranged from 1.75 to 65.38% (Fig. 6). The DCA curve demonstrated that the model provided a net clinical benefit across a wide range of threshold probabilities, from 1.75 to 65.38%. This indicates that for patients with a predicted POP risk within this range, using the model to guide clinical decision-making would yield a net benefit compared to either treating all patients or treating none. Threshold probabilities below 1.75% or above 65.38% are less common in clinical practice, suggesting that the model has practical utility for risk stratification in the majority of elderly hip fracture patients.

### 3.7. Clinical implementation: online risk calculator deployment

**Figure 7.**
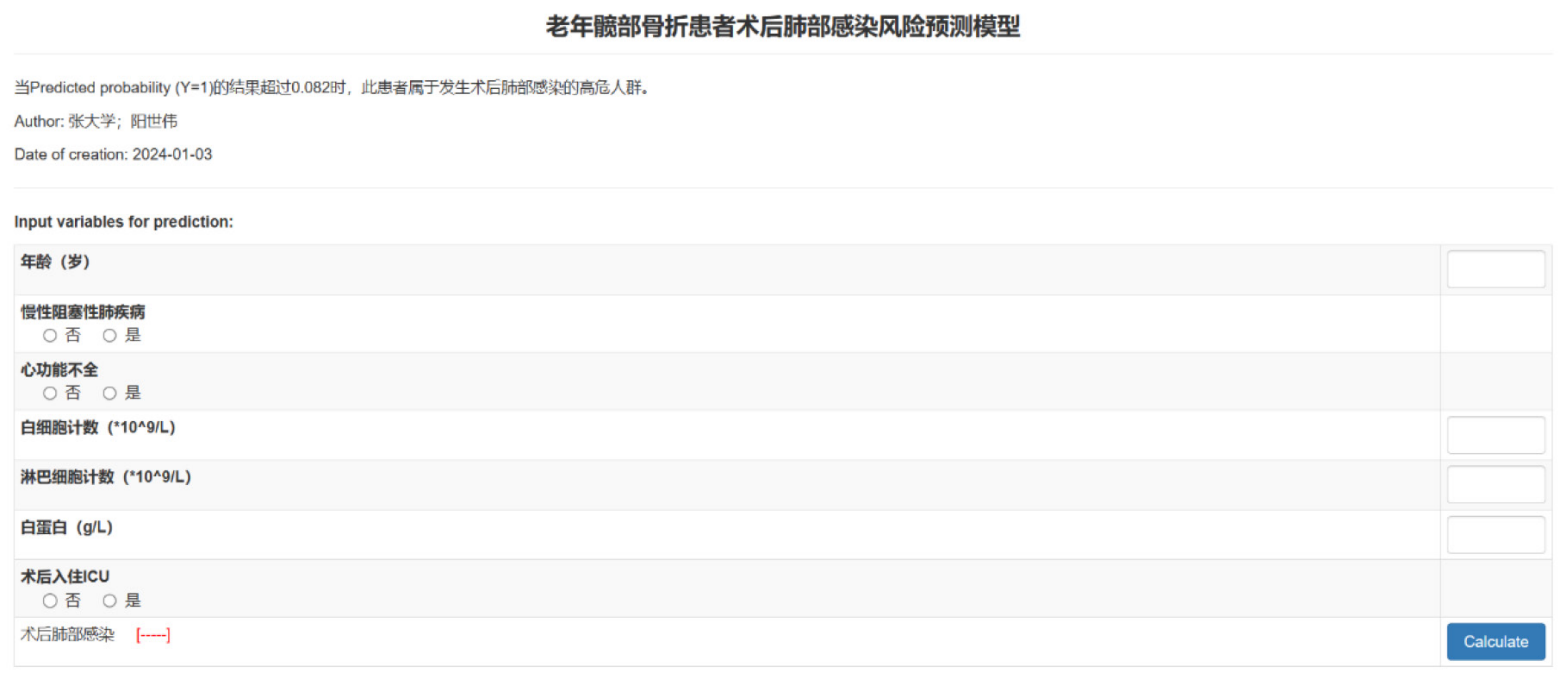
Screenshot of the online POP risk calculator developed based on data from 3 research centers. POP = postoperative pneumonia.

To enhance the clinical applicability of our predictive model for POP in elderly patients with hip fractures, we developed a user-friendly, web-based risk calculator. The calculator was constructed based on the final model derived from the data collected at 3 medical centers, and it enables individualized risk estimation for each patient based on their clinical parameters. As shown in Figure 7, users can input relevant patient data to obtain real-time predictions of the probability of POP. This tool was designed to assist clinicians in early risk assessment and in making informed decisions regarding perioperative care strategies. This calculator is freely accessible at http://www.empowerstats.net/pmodel/?m=34885_HFPOIPC.

## 4. Discussion

We included clinical data from elderly patients with hip fractures in 3 hospitals, and the results showed a POP incidence rate of 6.56% (64/976) in elderly hip fracture patients. Pneumonia is a serious complication of hip fracture, particularly in elderly patients. Despite strategies such as antibiotic treatment, promotion of postoperative coughing and sputum clearance, and perioperative pulmonary rehabilitation training, the risk of POP remains relatively high.^[16,17]^ Pneumonia is the most common perioperative complication and the leading cause of mortality after hip fractures in older adults. Therefore, identifying risk factors for POP in elderly patients with hip fractures and developing a risk prediction model are crucial for reducing the incidence and mortality rate of pneumonia, improving the quality of life of elderly patients after hip fracture surgery, and providing targeted prevention and treatment for these risk factors in future clinical practice.

Univariate analysis revealed that 23 variables were associated with POP. After applying LASSO regression, 17 variables were selected for inclusion in multivariate logistic regression analysis. The results revealed that advanced age, high WBC count, low lymphocyte count, low albumin level, COPD, HF, and postoperative ICU admission were independent risk factors for POP in elderly patients with hip fractures (all *P* < .05). Based on these independent risk factors, we constructed a nomogram predictive model for POP in elderly patients with hip fractures. This model demonstrated moderate discrimination (AUC = 0.795) and good calibration, enabling the identification of high-risk patients who were more likely to develop POP. We acknowledge that the multicenter nature of our study may introduce site-specific effects, and future external validation should assess model performance across different centers.

Our results identified age as an independent risk factor for POP in elderly patients with hip fractures (odds ratio [OR]: 1.059; 95% CI: 1.022–1.098). The main reason for this association is that as patients age, their immune function gradually weakens, leading to a decreased ability to defend against pathogens. Additionally, the pain caused by the fracture and the impact of surgical stress make older individuals more susceptible to various infections.^[18]^ Furthermore, as age increases, organ functions progressively decline, including changes in the respiratory system such as decreased chest wall compliance, weakened respiratory muscle strength, reduced ciliary clearance rate, and diminished cough reflex.^[19]^ However, studies conducted by Xiang et al,^[20]^ Zhang et al,^[21]^ and Ji et al,^[22]^ among others, did not find age to be an independent risk factor for hip fractures in elderly patients. The main reason for the controversy regarding age as a risk factor may be that hip fracture patients not only face the issue of advanced age but also often have comorbidities, especially respiratory diseases. Therefore, age alone is not an absolute contraindication for surgery in patients with hip fractures. After thorough patient assessment, even elderly patients can undergo surgical treatment to accelerate postfracture recovery.^[23]^

The results of this study showed that elderly hip fracture patients with comorbid COPD had a 3.9-fold higher risk of developing POP than patients without COPD. This indicates that COPD significantly increases the risk of POP. COPD patients typically have increased airway secretions and airflow limitation, which can lead to mucus retention and subsequently increase the risk of postoperative pulmonary complications.^[24,25]^ Additionally, other studies have reported that in patients undergoing hip arthroplasty, the presence of COPD is associated with at least a sevenfold increase in postoperative mortality rates.^[26]^ Similarly, our study also identified the presence of HF as an independent risk factor for POP in patients with hip fractures (OR: 3.057, 95% CI: 1.189–7.859). Related studies^[27,28]^ have also found an increased risk of POP in patients with concurrent HF. Impaired cardiac function can lead to alterations in systemic circulation and tissue metabolism, which in turn affect immune system function and decrease the body’s resistance to bacteria or other pathogens. Zhang et al,^[29]^ using an elderly rat model, found that after hip fracture surgery, rats experienced systemic inflammatory responses and lung injury, including inflammatory cell infiltration, pulmonary edema, congestion, and alveolar hemorrhage. Therefore, patients with concurrent HF are more likely to develop pneumonia after hip fractures.

WBC are a crucial component of the immune system, and changes in their levels often reflect the immune response of the body to infection or inflammation. An abnormal WBC count may indicate alterations in a patient’s immune status. The results of this study suggest that as the preoperative WBC count increases, the risk of POP in elderly patients with hip fractures also increases (OR: 1.135; 95% CI: 1.035–1.245). The main reason for this association is that WBCs infiltrate the interstitial space through endothelial gaps in capillaries and phagocytize pathogens and tissue debris. However, elevated WBC counts can reduce phagocytic activity, compromising the body’s defense mechanisms and increasing the risk of infection.^[30]^ Moreover, we found that lymphocytes, a subset of WBCs, were an independent influencing factor for POP in elderly hip fracture patients (OR: 0.469; 95% CI: 0.253–0.869). Related research^[31]^ has reported that lymphocytes can serve as predictors of adverse outcomes in elderly patients with hip fractures after surgery. Consistent with our findings, a study on community-acquired pneumonia found a negative correlation between lymphocytes and community-acquired pneumonia (*r* = −0.268, *P* = .018), suggesting that lymphocytes can be independent predictive factors.^[32]^

Although WBC is an important indicator of immune function, most studies have not found WBC to be a predictive factor for POP in elderly hip fracture patients.^[12,28,33,34]^ Although WBC are immune defense cells involved in various cellular immune responses and exhibit high sensitivity during stress reactions, their specificity is relatively low. Therefore, an increase in WBC count does not necessarily indicate an increased risk of pneumonia. Furthermore, especially when dealing with elderly individuals, WBC count may not be sensitive enough, and the rise in WBC count may not be significant when the body experiences an infection. It should be noted that although WBC count may not be a specific predictive factor for POP, it can still serve as one of the reference indicators for assessing the risk of postoperative infection. Therefore, it should be considered, along with other risk factors, as part of the overall judgment.

The results of this study demonstrated that preoperative low albumin levels were an independent risk factor for POP in elderly patients with hip fractures (OR: 0.920; 95% CI: 0.861–0.984). As a marker of malnutrition, nearly half of the elderly hip fracture patients have low serum albumin levels upon admission.^[35,36]^ Previous studies^[10,36]^ have indicated that preoperative malnutrition increases the incidence of POP. The main reason for this is that proper healing of the fracture site and muscle strength recovery requires an adequate supply of protein. Inadequate nutrition delays wound healing, leading to prolonged bed rest that increases the risk of pneumonia. Additionally, low serum albumin levels can result in immune dysfunction, thereby reducing the patient’s ability to resist infections. Decreased albumin levels also contribute to increased capillary permeability, allowing fluid to enter the interstitial space, leading to pleural effusion and pulmonary edema, and further increasing the risk of pneumonia.^[37]^

Patients transferred to the ICU after surgery have a 3.82-fold increased risk of developing pneumonia, which significantly affects their prognosis. The ICU is a concentrated area for critically ill patients where various pathogens and drug-resistant bacteria are densely distributed. Elderly hip fracture patients admitted to the ICU after surgery are usually in critical condition, with severely compromised immune function. Factors such as mechanical ventilation and invasive tracheal procedures further increase the incidence of POP in elderly patients.^[38,39]^ Therefore, special attention should be paid to this high-risk group in postoperative management, and preventive measures should be taken to reduce the occurrence of pulmonary infections.

### 4.1. Limitations

Several limitations of this study should be acknowledged. First, due to its retrospective design, the possibility of information bias cannot be completely excluded, although standardized data collection procedures were implemented across the 3 participating hospitals. Second, while our sample size of 976 patients is relatively large, the number of outcome events (64 cases of POP) was limited, resulting in an events-per-variable ratio of approximately 9. This is slightly below the recommended threshold of 10 and may increase the risk of model overfitting. Consequently, external validation in larger, independent cohorts is essential before clinical application. Third, the inclusion of postoperative ICU admission as a predictor introduces temporal ambiguity, as it occurs after surgery and may lie on the causal pathway to pneumonia. While this variable was included to enable dynamic postoperative risk assessment, we acknowledge that it may overestimate predictive performance. Future studies should consider developing separate models for preoperative and postoperative risk stratification. Fourth, continuous predictors were modeled as linear terms without assessing potential nonlinear relationships, and our calibration assessment was limited to calibration plots and the Hosmer–Lemeshow test; additional metrics such as calibration slope, calibration intercept, and Brier score were not calculated. These represent methodological trade-offs made to maintain model parsimony given the limited outcome events and should be addressed in future research with larger sample sizes. Fifth, our exclusion of patients with severe organ insufficiency and in-hospital mortality may limit generalizability to these high-risk populations and could potentially underestimate the true incidence of POP. Sixth, although data were collected from 3 hospitals in Shenzhen, China, all institutions are located in the same geographic region, which may restrict applicability to other healthcare settings with different patient demographics or perioperative protocols. Finally, POP was only assessed during hospitalization; post-discharge pneumonia events were not captured, which may underestimate the true burden of this complication. Extended follow-up in future studies would provide a more complete picture.

Despite these limitations, this multicenter study provides a clinically useful tool for identifying elderly hip fracture patients at elevated risk for POP, using readily available clinical variables. The model demonstrated moderate discrimination and good calibration in internal validation, and the online risk calculator facilitates its potential clinical application pending external validation.

## 5. Conclusion

The incidence of POP in elderly patients with hip fractures was 6.56%. Advanced age, high WBC count, low lymphocyte count, low albumin level, COPD and HF comorbidities, and postoperative ICU admission were identified as independent risk factors for POP in these patients. The predictive model for POP developed based on multicenter data demonstrated moderate discrimination and good calibration. However, external validation is required before clinical implementation.

## Acknowledgments

We greatly appreciate the financial support provided by Shenzhen Science and Technology Innovation Committee, Shenzhen Second People’s Hospital, and the School of Nursing, Anhui Medical University. We also sincerely acknowledge the support of Peking University Shenzhen Hospital and Shenzhen Pingle Orthopedic Hospital for providing access to clinical data.

## Author contributions

**Data curation:** Daxue Zhang, Jian Kang.

**Formal analysis:** Daxue Zhang.

**Funding acquisition:** Xuchun Li.

**Investigation:** Xuchun Li.

**Methodology:** Daxue Zhang, Jian Kang, Yongli Zhang, Shiwei Yang, Xuchun Li.

**Software:** Yongli Zhang.

**Writing – original draft:** Daxue Zhang.

**Writing – review & editing:** Shiwei Yang, Xuchun Li.
